# Supplementary material for: Who would take part in a pandemic preparedness cohort study? The role of vaccine-related affective polarisation: Cross-sectional survey
Source: PLoS One. 2026 Apr 20;21(4):e0346420. doi: 10.1371/journal.pone.0346420 (PMC13095020; doi:10.1371/journal.pone.0346420)
Supplement: S1 Fig — (PDF) [file pone.0346420.s001.pdf]

S1 figure: Distribution of raw data from variables which were dichotomised

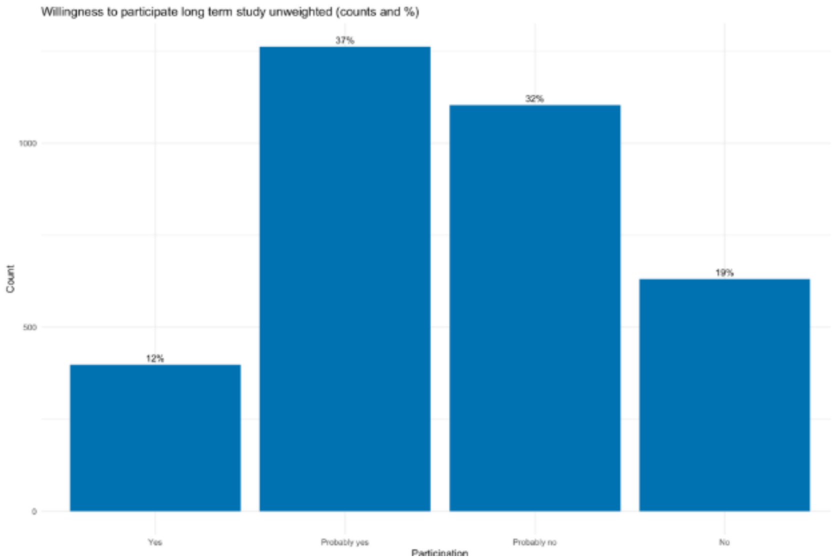

1a: Responses to, “In principle, would you be willing to participate in a long-term study?”

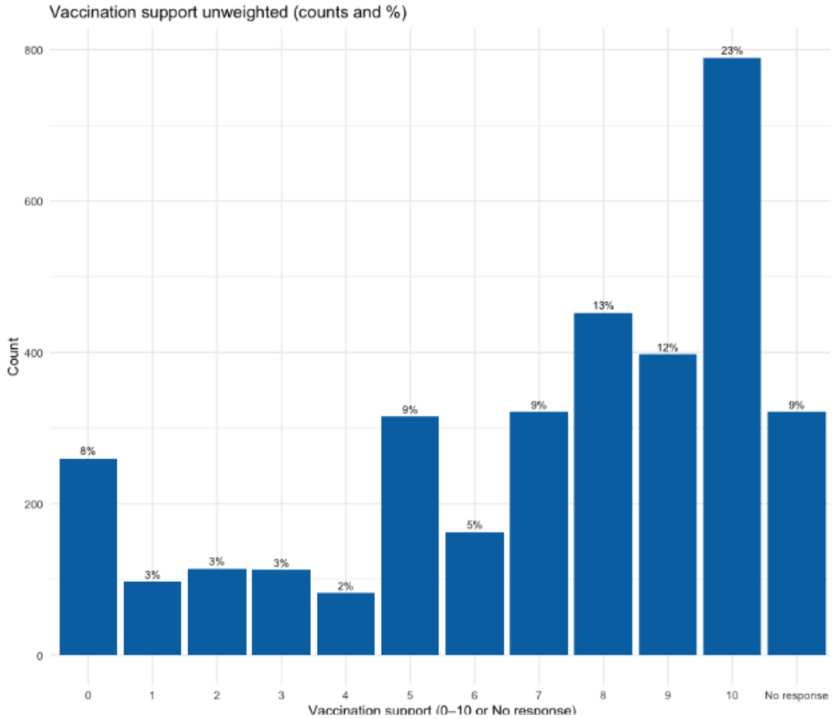

1b: Responses to “What do you think about vaccinations against COVID-19?”

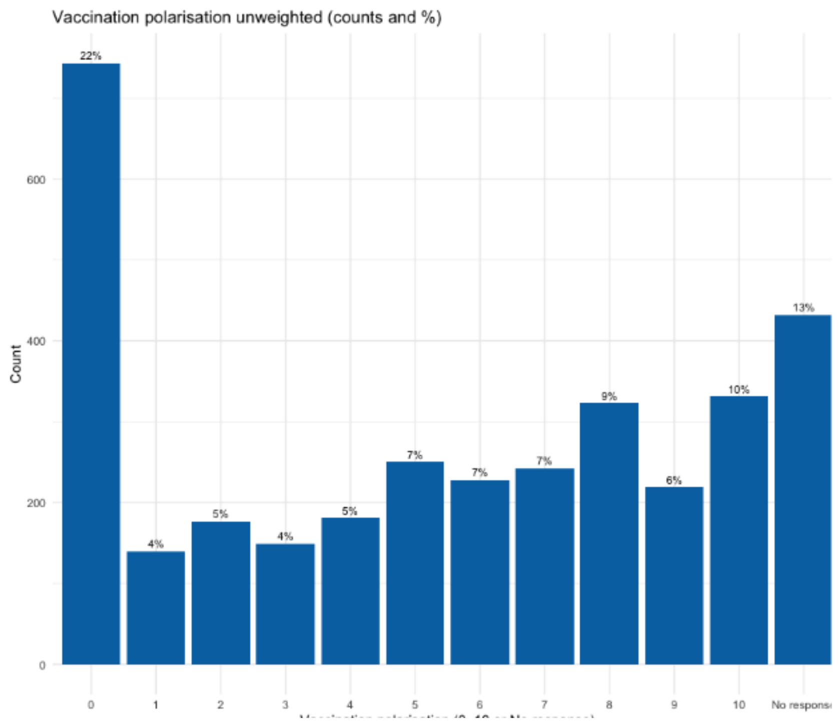

1c: Affective polarisation was calculated as the absolute difference between participants' feelings toward people who get vaccinated and those who do not, each rated on a scale from -5 (very negative) to +5 (very positive). The resulting score ranges from 0 to 10, where 0 indicates no polarisation and 10 indicates strong affective polarisation.
