# Supplementary material for: Who would take part in a pandemic preparedness cohort study? The role of vaccine-related affective polarisation: Cross-sectional survey
Source: PLoS One. 2026 Apr 20;21(4):e0346420. doi: 10.1371/journal.pone.0346420 (PMC13095020; doi:10.1371/journal.pone.0346420)
Supplement: S2 Fig — (PDF) [file pone.0346420.s002.pdf]

S2 figure: Study participants flow chart

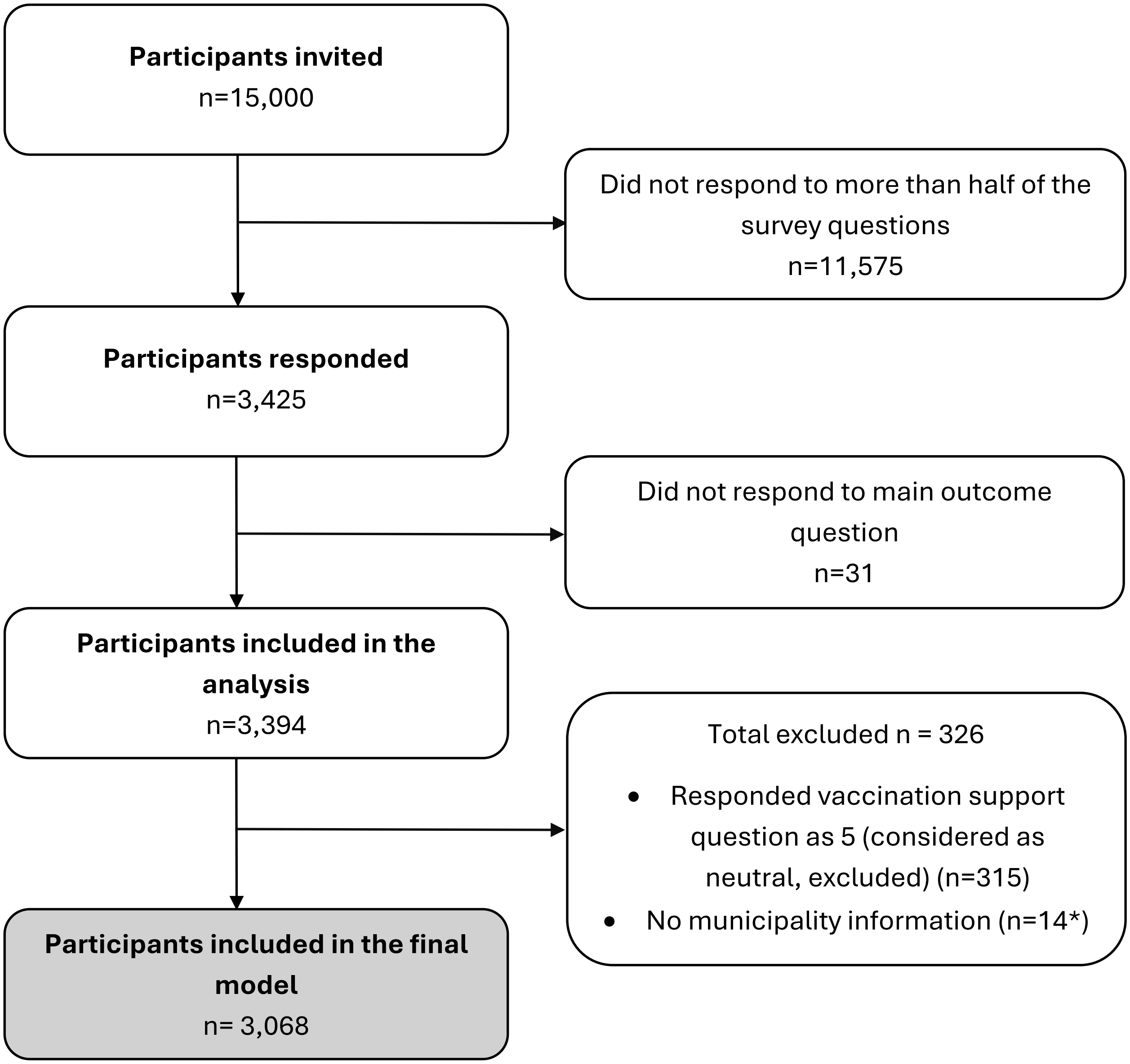

*3 missing values in "no municipality information" overlaps with responded vaccination as 5.
