# Supplementary material for: Who would take part in a pandemic preparedness cohort study? The role of vaccine-related affective polarisation: Cross-sectional survey
Source: PLoS One. 2026 Apr 20;21(4):e0346420. doi: 10.1371/journal.pone.0346420 (PMC13095020; doi:10.1371/journal.pone.0346420)
Supplement: S1 Table — (PDF) [file pone.0346420.s003.pdf]

S1 table: STROBE statement- Checklist of items that should be included in reports of *cross-sectional studies*

STROBE Statement—Checklist of items that should be included in reports of ***cross-sectional studies***

	Item No	Recommendation	Place in the article
**Title and abstract**	1	(*a*) Indicate the study's design with a commonly used term in the title or the abstract	Title
		(*b*) Provide in the abstract an informative and balanced summary of what was done and what was found	Abstract
**Introduction**			
Background/rationale	2	Explain the scientific background and rationale for the investigation being reported	Background, paragraph 1-2
Objectives	3	State specific objectives, including any prespecified hypotheses	Background, paragraph 2
**Methods**			
Study design	4	Present key elements of study design early in the paper	Methods, paragraph 1
Setting	5	Describe the setting, locations, and relevant dates, including periods of recruitment, exposure, follow-up, and data collection	Methods, paragraph 1<br>Sample, paragraph 1
Participants	6	(*a*) Give the eligibility criteria, and the sources and methods of selection of participants	Sample, paragraph 1
Variables	7	Clearly define all outcomes, exposures, predictors, potential confounders, and effect modifiers. Give diagnostic criteria, if applicable	Survey development, paragraph 1-2
Data sources/ measurement	8*	For each variable of interest, give sources of data and details of methods of assessment (measurement). Describe comparability of assessment methods if there is more than one group	Survey development, paragraph 1-2
Bias	9	Describe any efforts to address potential sources of bias	Sample, paragraph 1
Study size	10	Explain how the study size was arrived at	Sample, paragraph 1

Quantitative variables	11	Explain how quantitative variables were handled in the analyses. If applicable, describe which groupings were chosen and why	Statistical analysis, paragraph 1<br><br>Table S2
Statistical methods	12	(*a*) Describe all statistical methods, including those used to control for confounding	Statistical analysis
		(*b*) Describe any methods used to examine subgroups and interactions	Statistical analysis, paragraph 2
		(*c*) Explain how missing data were addressed	Table S2 and table S5
		(*d*) If applicable, describe analytical methods taking account of sampling strategy	NA
		(*e*) Describe any sensitivity analyses	Statistical analysis, paragraph 3<br><br>Table S6
**Results**			
Participants	13*	(a) Report numbers of individuals at each stage of study—eg numbers potentially eligible, examined for eligibility, confirmed eligible, included in the study, completing follow-up, and analysed	Figure S2
		(b) Give reasons for non-participation at each stage	Figure S2
		(c) Consider use of a flow diagram	Figure S2
Descriptive data	14*	(a) Give characteristics of study participants (eg demographic, clinical, social) and information on exposures and potential confounders	Sample description, paragraph 1-2
		(b) Indicate number of participants with missing data for each variable of interest	Table 1
Outcome data	15*	Report numbers of outcome events or summary measures	Table 1 and table S5
Main results	16	(*a*) Give unadjusted estimates and, if applicable, confounder-adjusted estimates and their precision (eg, 95% confidence interval). Make clear which confounders were adjusted for and why they were included	Statistical analysis, paragraph 3<br><br>Table 2

		(*b*) Report category boundaries when continuous variables were categorized	Table S2
		(*c*) If relevant, consider translating estimates of relative risk into absolute risk for a meaningful time period	NA
Other analyses	17	Report other analyses done—eg analyses of subgroups and interactions, and sensitivity analyses	Statistical analysis, paragraph 2-3
**Discussion**			
Key results	18	Summarise key results with reference to study objectives	Discussion, paragraph 1
Limitations	19	Discuss limitations of the study, taking into account sources of potential bias or imprecision. Discuss both direction and magnitude of any potential bias	Discussion, paragraph 2
Interpretation	20	Give a cautious overall interpretation of results considering objectives, limitations, multiplicity of analyses, results from similar studies, and other relevant evidence	Discussion, paragraph 2-5
Generalisability	21	Discuss the generalisability (external validity) of the study results	Discussion, paragraph 3 & 5
**Other information**			
Funding	22	Give the source of funding and the role of the funders for the present study and, if applicable, for the original study on which the present article is based	Footnotes, paragraph 2

*Give information separately for exposed and unexposed groups.

Note: An Explanation and Elaboration article discusses each checklist item and gives methodological background and published examples of transparent reporting. The STROBE checklist is best used in conjunction with this article (freely available on the Web sites of PLoS Medicine at http://www.plosmedicine.org/, Annals of Internal Medicine at http://www.annals.org/, and Epidemiology at http://www.epidem.com/). Information on the STROBE Initiative is available at www.strobe-statement.org.
