# Supplementary material for: Who would take part in a pandemic preparedness cohort study? The role of vaccine-related affective polarisation: Cross-sectional survey
Source: PLoS One. 2026 Apr 20;21(4):e0346420. doi: 10.1371/journal.pone.0346420 (PMC13095020; doi:10.1371/journal.pone.0346420)
Supplement: S2 Table — (PDF) [file pone.0346420.s004.pdf]

S2 table: Descriptive table for variables included in the analysis

Variable Name	Survey Question Asked	Survey answers	Analysis
Households size	Number of people in your household (including yourself)	1 to 20	Categorical variable<br>1<br>2<br>3<br>4<br>5 or more
Education level	What is your highest level of education?	1) Primary school<br>2) Secondary school 3) Grammar school 4) Vocational training/Federal Vocational Baccalaureate<br>5) University degree: Bachelor<br>6) University degree: Master/Diploma<br>7) University degree: Doctorate/PhD<br>8) Other	Categorical variable<br>Compulsory or less (1 and 2)<br>Upper secondary (3 and 4)<br>Tertiary education (5, 6 and 7)<br>Other (8)<br>No response
Age in years	Age person	18 to 120 years	Continuous variable
House location	Where is your flat/house located?	1) City centre<br>2) Suburb<br>3) Industrial area<br>4) Rural area<br>5) Adjacent to farmland<br>6) Other	Categorical variable<br>City centre (1)<br>Suburb (2)<br>Rural area (4 and 5)<br>Other (3 and 6)
Income, Swiss Francs	What is your average net household income per month?	1) <3,000<br>2 3,000-4,500<br>3) 4,500-6,000<br>4) 6,000-9,000<br>5) 9,000-11,000<br>6) 11,000 or more<br>7) Prefer not to say<br>8) Don't know	Categorical variable<br><4,500 (1 and 2)<br>4,500-9,000 (3 and 4)<br>>9,000 (5 and 6)<br>Other (7 and 8)<br>No response
Current work situation	How would you best describe your current labour situation?	1) Working full-time (80%+)<br>2) Full-time housewife/-husband (80% or more)<br>3) Working part-time (<80%)<br>4) Casual worker<br>5) Unemployed<br>6) Never worked<br>7) In education<br>8) Long-term absence, i.e. more than 3 months (unpaid leave, maternity leave etc.)<br>9) Not working due to health reasons (incl. Disability benefits)<br>10) Pensioner<br>11) Pensioner but working (incl. in an honorary capacity)<br>12) Volunteer	Categorical variable<br>Full time employee (1)<br>Part time employee (3,4 and 11)<br>Not employed (2, 5, 6,8,9,10 and 12)<br>In education (7)<br>Other (13)<br>No response

		13) Other	
Gender	I am ...	1) Male<br>2) Female<br>3) Non-binary<br>4) Prefer not to say<br>5) Don't know	Categorical variable<br>Female (2)<br>Male (1)<br>Other (3,4 and 5)<br>No response
Language	Languages	1) Deutsch<br>2) Français<br>3) Italiano<br>4) English	Categorical variable<br>German<br>French<br>Italian<br>English
Nationality	What's your nationality?	1) Swiss<br>2) Foreign nationality<br>3) More than one nationality<br>4) Prefer not to say<br>5) Don't know	Categorical variable<br>Swiss<br>Foreign<br>No response
Feelings about COVID-19 vaccination	What do you think about vaccinations against COVID-19?	0 to 10<br>(0, I completely reject them to 10, I completely support them)<br>Slider used to set response	Categorical variable<br>Vaccination supporter (>5)<br>Vaccination opposer (<5)<br>(5 neither supporter or opposer)
Feeling thermometer questions	Some people do not want to get vaccinated while others get vaccinated against COVID-19. Now, please think about these two groups. How do you feel about people belonging to these two groups? Please describe your feelings using a thermometer ranging from-5 to +5. -5 means that you are very cold and negative towards the group. +5 means that you are very sympathetic and positive towards the group.		
Feelings about people who get vaccinated	People who get vaccinated	-5 to +5<br>-5 very cold and negative<br>+5 very warm and positive<br>Change the slider above to set a response.	Not directly part of the model<br>"Vaccination related affective polarisation" variable calculated with this variable.
Feelings about people who do not get vaccinated	People who do not get vaccinated	-5 to +5<br>-5 very cold and negative<br>+5 very warm and positive<br>Slider used to set response	Not directly part of the model<br>"Vaccination related affective polarisation" variable calculated with this variable.
Vaccination related affective polarisation (Calculated)	Not a survey question calculated using "Feelings about people who get vaccinated" and "Feelings about people who do not get vaccinated"	Not a survey question calculated using "Feelings about people who get vaccinated" and "Feelings about people who do not get vaccinated"	Binary variable<br>Polarised<br>Not polarised
Willingness to participate long term study	In principle, would you be willing to participate in a long-term study	1) Not at all<br>2) Yes, definitely<br>3) Rather yes<br>4) Rather no	Binary variable (Outcome)<br>Yes (2 and 3)<br>No (1 and 4)

What would be your motivation(s) to participate in a long-term study and to donate time, information and potentially biological samples to it? (responders can choose multiple answers from down below)
In this way I can contribute to the health of fellow human beings
I can contribute to better preparation for the next pandemic
I am interested in research and health
I get a free health check
I receive the study results as feedback
It makes me proud to be a participant in an important cantonal study
Other reasons (free text option)

For what reasons would you refuse participation in a long-term study?	Assigned categories in figure 2
I am not interested in it	Not interested
I don't think much of health research	Not interested
I don't want to share my health data	Privacy concerns
I am worried that my data might not be sufficiently well protected	Privacy concerns
I am worried that my data may be abused (e.g. by health insurers, employers or similar)	Privacy concerns
I don't want to donate blood or other biological samples	Mistrust
I am worried that my voluntary contribution may serve the private interests of the pharmaceutical industry	Mistrust
I don't believe that such a study will improve the health of the population	Mistrust
I have no time	No time
I only have time in the evening and on weekends to participate in a study	No time
I don't want to visit a study centre	No benefit
I don't personally benefit from the results of such a study	No benefit
Prefer not to say	Other reasons / no response
Other reasons	Other reasons / no response
Don't know	Other reasons / no response
