# Supplementary material for: Who would take part in a pandemic preparedness cohort study? The role of vaccine-related affective polarisation: Cross-sectional survey
Source: PLoS One. 2026 Apr 20;21(4):e0346420. doi: 10.1371/journal.pone.0346420 (PMC13095020; doi:10.1371/journal.pone.0346420)
Supplement: S3 Table — (PDF) [file pone.0346420.s005.pdf]

S3 table: Weighting levels and represented households

We applied weighting to our dataset at two different levels. At the first level, we weighted all invited participants to conduct a descriptive analysis comparing responders and non-responders. At the second level, we weighted only the participants who responded in order to perform our primary logistic regression analysis.

Level 1: Invited participants			Level 2: Responded participants		
Household size	Study sample (invited)	Households represented	Household size	Study sample (respondents)	Households represented
1	3,000	185,820	1	639	185,820
2	3,000	170,629	2	877	170,629
3	3,000	54,814	3	718	54,814
4	3,000	53,981	4	809	53,981
5+	3,000	24,729	5+	351	24,729
