# Supplementary material for: Who would take part in a pandemic preparedness cohort study? The role of vaccine-related affective polarisation: Cross-sectional survey
Source: PLoS One. 2026 Apr 20;21(4):e0346420. doi: 10.1371/journal.pone.0346420 (PMC13095020; doi:10.1371/journal.pone.0346420)
Supplement: S4 Table — (PDF) [file pone.0346420.s006.pdf]

S4 table: Responders of the survey by household size and language

	Response to survey								
	Yes			No			Total		
	N[1]	Col%[2]	Row%[3]	N[1]	Col%[2]	Row%[3]	N[1]	Col%[2]	Row%[3]
**Household size**									
1	639	18.8	21.3	2,361	20.3	78.7	3,000	20	100
2	877	25.8	29.2	2,123	18.3	70.8	3,000	20	100
3	718	21.2	23.9	2,282	19.7	76.1	3,000	20	100
4	809	23.8	27.0	2,191	18.9	73.0	3,000	20	100
5+	351	10.3	11.7	2,649	22.8	88.3	3,000	20	100
**Language**									
German	3,074	90.6	22.8	10,400	89.6	77.2	13,474	89.8	100
French	312	9.2	21.2	1,161	10.0	78.8	1,473	9.8	100
Italian	2	0.06	18.2	9	0.08	81.8	11	0.07	100
English	6	0.17	14.6	35	0.30	85.4	41	0.23	100

[1] Number of observations
[2] Column percentage
[3] Row percentage
