# Supplementary material for: Who would take part in a pandemic preparedness cohort study? The role of vaccine-related affective polarisation: Cross-sectional survey
Source: PLoS One. 2026 Apr 20;21(4):e0346420. doi: 10.1371/journal.pone.0346420 (PMC13095020; doi:10.1371/journal.pone.0346420)
Supplement: S5 Table — (PDF) [file pone.0346420.s007.pdf]

S5 table: Willingness to participate in a long-term cohort study, unweighted denominators and proportions

		Denominator	Willingness to participate long term study
**Responders**		3,394	1,660
**Age, years (SD; range)**		3,394	48.6 (16.5; 18-94)
**Age group, years**	18-29	404 (11.9%)	223 (55.2%)
	30-64	2,261 (66.6%)	1,132 (50.0%)
	64+	729 (21.4%)	305 (41.8%)
**Gender**	Female	1,787 (52.7%)	899 (50.3%)
	Male	1,580 (46.6%)	750 (47.5%)
	Other	13 (0.4%)	7 (53.8%)
	No response	14 (0.4%)	4 (28.6%)
**Education level**	Compulsory education or less	488 (14.4%)	153 (31.4%)
	Upper secondary	1,522 (44.8%)	668 (43.9%)
	Tertiary	1,300 (38.3%)	808 (62.2%)
	Other	26 (0.8%)	10 (38.5%)
	No response	58 (1.7%)	21 (36.2%)
**Current work situation**	Full-time employee	1,473 (43.4%)	770 (52.3%)
	Part-time employee	815 (24%)	439 (53.9%)
	Not employed	830 (24.5%)	336 (40.5%)
	In education	127 (3.7%)	73 (57.5%)
	Other	56 (1.6%)	19 (33.9%)
	No response	93 (2.7%)	23 (24.7%)
**Income, Swiss Francs**	<4,500	518 (15.3%)	213 (41.1%)
	4,500 – 9,000	1,349 (39.8%)	666 (49.4%)
	>9,000	911 (26.8%)	583 (64.0%)
	Other	480 (14.1%)	161 (33.5%)
	No response	136 (4.0%)	37 (27.2%)
**Household size**	1	639 (18.8%)	336 (52.6%)
	2	877 (25.8%)	423 (48.2%)
	3	718 (21.2%)	367 (51.1%)
	4	809 (23.8%)	392 (48.5%)
	5+	351 (10.3%)	142 (40.5%)
**Household location**[1]	Urban	1,864 (54.9%)	970 (52.0%)
	Intermediate	861 (25.4%)	405 (47.0%)
	Rural	655 (19.2%)	279 (42.6%)
**Nationality**	Swiss	2,716 (80.0%)	1,377 (50.7%)

	Foreign	637 (18.8%)	273 (42.9%)
	No response	41 (1.2%)	10 (24.4%)
**Language**	German	2,922 (86.1%)	1,435 (49.1%)
	French	330 (9.7%)	154 (46.7%)
	Italian	49 (1.4%)	22 (44.9%)
	English	93 (2.7%)	49 (52.7%)
**Vaccination support status[2]**	For vaccination	2,106 (62.0%)	1,220 (57.9%)
	Against vaccination	655 (19.3%)	210 (32.1%)
	No response	321 (9.5%)	102 (31.8%)
**Vaccination related affective polarisation**	Polarised	1,376 (40.5%)	607 (44.1%)
	Not polarised	1,586 (46.7%)	893 (56.3%)
	No response	432 (12.7%)	160 (37.0%)
	**Willingness to participate with children**		
		**Number with children**	**Willingness to participate**
			Yes
**On behalf of their children**	-	1,083	450 (41.6%)
	**Willingness to participate with pets**		
		**Number with pets**	**Willingness to participate**
			Yes
**On behalf of their pets**	Any pet	1,066	594 (55.7%)
**Type of Pet[3]**	Dogs	336 (31.5%)	158 (47%)
	Cats	710 (66.6%)	312 (43.9%)
	Rabbits	83 (7.8%)	34 (41%)
	Rodents	71 (6.6%)	38 (53.5%)
	Others	124 (11.6%)	56 (45.2%)

[1] The sum of percentages for the household location variable does not equal 100% due to missing data that were not classified (n=14) by the Cantonal Administration and Information Office.

[2] The sum of percentages for the opinions on vaccination does not total 100% because responses marked as “5” (n=312) - representing a neutral position at the midpoint of the scale - were excluded from the analysis.

[3] The sum of the percentages exceeds 100% because some households reported owning multiple types of pets.
