# Supplementary material for: Who would take part in a pandemic preparedness cohort study? The role of vaccine-related affective polarisation: Cross-sectional survey
Source: PLoS One. 2026 Apr 20;21(4):e0346420. doi: 10.1371/journal.pone.0346420 (PMC13095020; doi:10.1371/journal.pone.0346420)
Supplement: S6 Table — (PDF) [file pone.0346420.s008.pdf]

S6 table: Sensitivity analysis

	Multivariable (final) model		Sensitivity analysis model 1		Sensitivity analysis model 2		Sensitivity analysis model 3	
**Characteristic**	**OR[1]**	**95% CI[1]**	**OR[1]**	**95% CI[1]**	**OR[1]**	**95% CI[1]**	**OR[1]**	**95% CI[1]**
**Age, per year**	0.99	0.98 - 0.99	0.99	0.98 - 0.99	0.99	0.98 - 0.99	0.99	0.98 - 0.99
**Education level**								
Compulsory education or less	1	1	1	1	1	1	1	1
Upper secondary education	1.59	1.19 - 2.12	1.53	1.16 - 2.02	1.54	1.17 - 2.03	1.59	1.19 - 2.12
Tertiary education	2.48	1.81 - 3.39	2.41	1.79 - 3.24	2.39	1.77 - 3.23	2.46	1.80 - 3.37
Other	1.57	0.49 - 5.05	1.36	0.49 - 3.77	1.54	0.55 - 4.35	1.56	0.48 - 5.07
No response	1.61	0.75 - 3.43	1.41	0.68 - 2.91	1.43	0.71 - 2.89	1.62	0.76 - 3.47
**Income, Swiss Francs**								
<4'500	1	1	1	1	1	1	1	1
4'500 - 9'000	1.21	0.93 - 1.59	1.18	0.92 - 1.53	1.18	0.91 - 1.52	1.22	0.94 - 1.60
>9'000	1.92	1.39 - 2.65	1.74	1.28 - 2.36	1.75	1.29 - 2.39	1.96	1.41 - 2.70
Other	0.62	0.44 - 0.87	0.60	0.43 - 0.83	0.61	0.44 - 0.85	0.62	0.44 - 0.87
No response	0.46	0.26 - 0.83	0.56	0.33 - 0.94	0.57	0.33 - 0.98	0.46	0.26 - 0.82
**Number of household members**								
1	1	1	1	1	1	1	1	1
2	0.74	0.59 - 0.95	0.75	0.59 - 0.94	0.75	0.60 - 0.95	0.74	0.58 - 0.95
3	0.65	0.50 - 0.85	0.69	0.53 - 0.89	0.67	0.52 - 0.87	0.65	0.49 - 0.85
4	0.50	0.38 - 0.67	0.55	0.42 - 0.71	0.54	0.42 - 0.71	0.51	0.38 - 0.67
5≤	0.67	0.44 - 1.01	0.66	0.45 - 0.98	0.66	0.45 - 0.97	0.66	0.43 - 0.99
**Opinion about vaccination**								
Support vaccination	1	1	1	1	1	1	1	1
Oppose vaccination	0.53	0.39 - 0.72	0.61	0.45 - 0.81	0.55	0.42 - 0.72	0.52	0.38 - 0.73
No response	0.34	0.07 - 1.69	0.39	0.08 - 1.83	0.34	0.07 - 1.60	0.55	0.11 - 2.82
**Vaccination polarisation**								
Not polarised	1	1	1	1	1	1	1	1
Polarised	1.51	1.20 - 1.89	1.69	1.38 - 2.07	1.52	1.22 - 1.91	1.48*	1.15 - 1.92
No response	0.88	0.47 - 1.67	1.11	0.63 - 1.95	0.89	0.47 - 1.68	0.89	0.47 - 1.68

**Polarisation x Oppose to vaccination[3]**	1	1	1	1	1	1	1	1
Opposed to vaccination * Polarised	0.33	0.19 - 0.57	0.29	0.17 - 0.50	0.41	0.25 - 0.66	0.28	0.14 - 0.57

[1] OR, odds ratio, CI, confidence interval

Multivariable (final) model is the main model that we presented in our manuscript, includes following variables: Age, gender, education level, current work situation, income, household size, household location, language, nationality, opposition to vaccination, polarisation:vaccination opposer(interaction term) and willingness to participate (Outcome). And vaccination support == 5 considered as neutral and excluded

Sensitivity analysis model 1: Includes same variables as the final model however vaccination support == 5 coded as vaccine support instead of being excluded

Sensitivity analysis model 2: Includes same variables as the final model however vaccination support == 5 coded as vaccine oppose instead of being excluded

Sensitivity analysis model 3: Includes same variables as the final model however affective polarisation coded as (≤3 = not polarised, 4 to 6 polarised, 7 and more heavily polarised)

*OR of heavily polarised group (7 and more)
