# Supplementary material for: Who would take part in a pandemic preparedness cohort study? The role of vaccine-related affective polarisation: Cross-sectional survey
Source: PLoS One. 2026 Apr 20;21(4):e0346420. doi: 10.1371/journal.pone.0346420 (PMC13095020; doi:10.1371/journal.pone.0346420)
Supplement: S7 Table — (PDF) [file pone.0346420.s009.pdf]

S7 table: Reasons to participate or decline long-term study, unweighted

**Reasons to participate long-term study**	
In this way I can contribute to the health of fellow human beings	1,559 (56%)
I can contribute to better preparation for the next pandemic	1,134 (41%)
I am interested in research and health	1,031 (37%)
I get a free health check	713 (26%)
I receive the study results as feedback	682 (25%)
It makes me proud to be a participant in an important cantonal study	209 (8%)
Other reasons	115 (4%)
**Reasons to decline participating long-term study**	
I am not interested in it	266 (42%)
I have no time	209 (33%)
I don't want to share my health data	133 (21%)
I don't want to donate blood (or other biological samples)	98 (16%)
I don't want to visit a study centre	81 (13%)
Prefer not to say	78 (12%)
I am worried that my voluntary contribution may serve the private interests of the pharmaceutical industry	77 (12%)
I don't believe that such a study will improve the health of the population	76 (12%)
I am worried that my data might not be sufficiently well protected	74 (12%)
I am worried that my data may be abused (e.g. by health insurers, employers or similar)	64 (10%)
Other reasons	45 (7%)
I don't personally benefit from the results of such a study	27 (4%)
Don't know	27 (4%)
I don't think much of health research	15 (2%)
I only have time in the evening and on weekends to participate in a study	12 (2%)

People who answered to reasons to participate question are those who answered “yes”, “rather yes” and “rather no” to willingness to participate long-term study question.
People who answered to reasons to decline participation question are those who answered “no” to willing to participate long-term study question.
The percentage totals exceed 100% because respondents could select multiple answers for these questions.
